# Could nodding syndrome in Northern Uganda be a form of autism spectrum disorder? an observational study design

**DOI:** 10.11604/pamj.2018.30.115.13634

**Published:** 2018-06-12

**Authors:** Denis Anywar Arony, Suzanne Gazda, David Lagoro Kitara

**Affiliations:** 1 Gulu University, Faculty of Medicine, Department of Biochemistry, Gulu, Uganda; 2 Founding President for Hope for HumaNs (HfH), Neurologist at the St Antonio, Texas, USA; 3 Gulu University, Faculty of Medicine, Department of Surgery, Gulu, Uganda

**Keywords:** Nodding syndrome, Gulu university, IDPs, autism spectrum disorder, metabolic disorder

## Abstract

**Introduction:**

Nodding syndrome (NS) is associated with high anion gap, biotinidase and acetyl carnitine deficiency, vitamin B6 and D deficiency and internal displacement. The objective of this study was to conduct a metabolic analysis on NS children and review literature on its similarities with ASD.

**Methods:**

We conducted biochemical analysis on blood and urine of NS children at Hope for HumaNs (HfH) centre in 2014 and reviewed literature on its similarities with ASD. Ethical approval was obtained from an IRB. Data analysis was conducted using STATA version 12 and a p-value less than 0.05 was considered significant.

**Results:**

We found biotinidase deficiency in NS with a mean 1.98 95% CI(1.61, 2.34; p < 0.001); Acetyl carnitine deficiency 16.92 95% CI(16.10,17.75; p<0.001); Low BMI-for-age 16.92 95% CI(16.10,17.75; p = 0.42); Age 14.08 95% CI(0.78,4.660; p = 0.007); IDP duration 4.82 95% CI(4.48, 5.21; p = 0.92); Age at NS onset 8.02 95% CI(7.03, 9.01; p = 0.001); NS associated with multiple nodding episodes (χ^2^)=22.15, p=0.005; NS siblings with NS (χ^2^) = 9.68, p = 0.004; NS were in IDPs (χ^2^) = 22.15, p = 0.005.

**Conclusion:**

These findings are indicative that NS is associated with biotinidase and acetyl carnitine deficiency, IDPs, and environmental exposures. There are no new cases of NS reported by Ugandan MOH and WHO since 2012 when the IDP camps were disbanded and communities resettled in their own communities and feed on their own grown foods. Perhaps NS may be akin to Autism Spectrum Disorder (ASD). This finding will help support all efforts towards the treatment and rehabilitation of NS children.

## Introduction

Autism Spectrum Disorders (ASDs) are a group of behaviorally defined neurodevelopmental disorders with lifelong consequences [1]. They are defined by impairments in communication and social interaction along with restrictive and repetitive behaviors [1]. ASD is now estimated to affect 1 out of 68 individuals in the United States with approximately four times more males than females affected [2]. Although ASD is behaviorally defined, children with ASD also have many co-occurring medical conditions such as gastrointestinal abnormalities [3] seizures and epilepsy [4] attention deficits [5] anxiety [6] and allergies [7]. One of its most significant co morbidities that causes significant disability is epilepsy [8]. In addition, a number of studies suggest that epilepsy affects a high proportion of individuals with ASD and a number of risk factors for autism can be categorized as risk factors for inflammation or indicators of inflammation [8]. Meanwhile, Nodding Syndrome (NS) is a new childhood neurological disorder characterized by atonic seizures, cognitive decline, muscle weakness, thermal dysfunction, internal displacement into IDPs, wasting, stunted growth and a number of repetitive behavioral abnormalities [9-11]. Recent case control study, case series and case reports conducted in Uganda identified high anion gap metabolic acidosis among NS children compared to their sex-and-age matched controls [9-11]. This researcher avers that nodding episodes are precipitated by sights of local food, starvation, exposure to cold weather/temperatures or cold water, physical exercises and there is an association with high anion gap [9,12]. In another study, there was an association with serum biotinidase and Acetyl carnitine deficiencies [9-12]. Additionally, other studies had observed a deficiency in Vitamin D [10-12]. These findings may perhaps suggest that NS could be secondary to a metabolic disorder and perhaps a mitochondrial disorder [9,11-13]. In addition, recent data on NS suggests an association with cerebrospinal fluid (CSF) VGKC antibodies and serum leiomidin-1 antibody, suggesting a neuro-inflammatory cause [14]. Furthermore, there is a demonstrated association with vitamin B6 deficiency [15]. The objective of this study was to conduct a biochemical analysis on urine and blood of NS children and review literature on its similarities with ASD.

## Methods

**Study design**: This was an observational study conducted on NS children admitted to Hope for HumaNs (HfH) centre situated in Odek, an area in the epicentre of NS epidemic in Northern Uganda [9,11,12].

Study site: This study was conducted in a largely rural community which has one of the highest levels of poverty, inadequate water and sanitation and with significant disease burden [9-12]. From 1986 to 2007/2008, this area was in civil war between the Ugandan Army and Lord's Resistance Army (LRA) [12]. Although the war raged on, the population were not displaced into IDP camps and continued to feed on their home grown foods. Interestingly, there were no reported cases of NS in the area from 1986 to 2001. However, in 2002 when the community had been IDPs for one year where they depended on food aid supplied by relief agencies, cases of NS appeared [9,11-13].The IDPs became associated with malnutrition, social norm breakdown, rising incidence of alcoholism, mental health disorders, febrile illnesses, suicidal tendencies, increasing prevalence of infectious diseases, neglect and waste of the youths [10,12,13]. After 2007, when the rebels were driven-out, the Ugandan Government began returning the IDPs to their homes in a phase-wise approach from the main camp to the satellite camps near their villages [12]. Eventually the communities were returned to their original home after extensive demining in the farmland where the returnees were to settle and rebuild their communities and lives [10,12]. In 2009, the Ugandan MOH identified NS and established screening and rehabilitation centres in 2012 where NS children were treated with anticonvulsants, multivitamins and nutritional supplements [10-14]. In 2012, HfH NS rehabilitation centre was established as a private initiative to complement the efforts of Government [12]. The centre was built in Aromowang lobo with classrooms for teaching basic education; medical clinic for treatment; a refectory and cooking place for food rehabilitation, a play field for soccer; a piggery for livelihood project and a medical staff quarter [12]. There was a daily schedule of activities for NS children beginning with travel from home, registration, administration of medication, physical exercises, feeding, bathing, hygiene training and physiotherapy [9,11,12].

**Study population**: We observed NS children who were undergoing outpatient rehabilitation at HfH centre and others were part of the outreach services of the centre, Ugandan MOH and Gulu District Health Department. Each child was individually screened and examined by the research team to conform to the inclusion criteria (probable NS) [9,11,12]**. Data collected** from individual NS child was extensive including the history of the syndrome and then a comprehensive clinical examination of each child.

**Recruitment methods**: We recruited the children for the study consecutively.

**Inclusion criteria**: The participants were recruited in accordance with WHO surveillance case definition of probable NS [9-12]. Informed consent from parents/guardians and assent for children 14 years and above were obtained.

**Exclusion criteria**: We excluded children 2 years and below and those with reported history of abnormal physical, cognitive and social development prior to onset of nodding.

**The study instruments**: A questionnaire was used to investigate the current and past physiological, psychosocial and mental health conditions of NS children. These questions were directed towards the parents/guardians and included information socio-demographic characteristics, when, where and how nodding episodes were first observed, the birth order, the relationship between onset and IDPs; food eaten in IDP, the weaning and complementary feeds for the NS Children, the trigger factors for nodding and the number of nodding episodes that occurred per day over the period [12].

**Anthropometric measurements**: Each NS child was measured clothed and barefoot for height (cm) and body weight (Kg). Weight was measured using a calibrated digital scale which was standardized before use while height was measured in centimeters using a stadiometer. The Mid-Upper-Arm-Circumference of the left arm was measured using a MUAC tape for the assessment of nutritional status and findings recorded in centimeters (cm).

**Ethical considerations**: This study was approved by a local IRB (LHIREC No. 065/10/14). The research team worked in collaboration with the administration of HfH centre, Gulu District Health Department and local councilors. Parents/guardians of NS children gave informed consent on behalf of the participants but for those above 14 years but below 18 years, assent was obtained. Two medical students from Gulu University were research assistants (Dr. Sarah and Dr. Lucy) together with a senior clinician DLK (author) supervised data collection. Most parents/guardians could not read or write and so we used the placement of inked thumbprints on the position for signature in the questionnaires. Furthermore, informed consent was obtained to allow the researchers publish these findings in international medical journals.

**Data analysis**: This was performed using STATA version 12 (Stata Corp LP, Texas, USA) where parametric data was presented as mean ± Standard Deviation (SD), maximum and minimum values. We used two medical students to extract information from the questionnaires independently and we compared the data consistency and resolved any inconsistencies with mutual agreement in consultation with the Principal Investigator. Conditional Logistic Regression was used to screen 74 potential explanatory variables (5 continuous, 45 ordinal and 24 dichotomous) for the associations with NS. Formal adjustments for the multiple testing were done to identify the associations and correlations with pre-specified lists of targets. We used Chi Square and Fisher's exact tests for bivariate analysis to identify differences between variables. We fitted ordinary least squares (OLS) regression models to identify trends in the variables in relation to NS. Stepwise regression was used as an exploratory tool to guide the introduction of covariates in our modeling approach. Finally, a multivariable logistic regression was conducted to identify the variables that correlated with the occurrence of nodding. A p-value less than 0.05 was considered significant.

## Results

**Figure 1:**
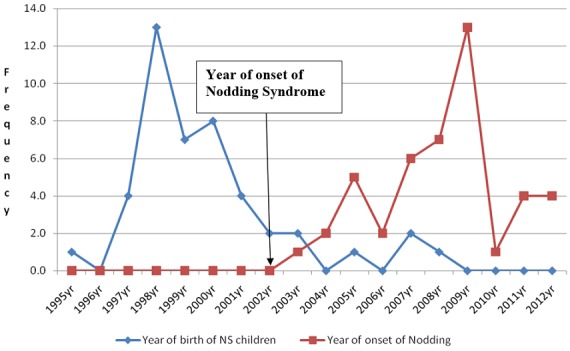
Year of birth and onset of nodding (Kitara et al, 2017)

**Figure 2:**
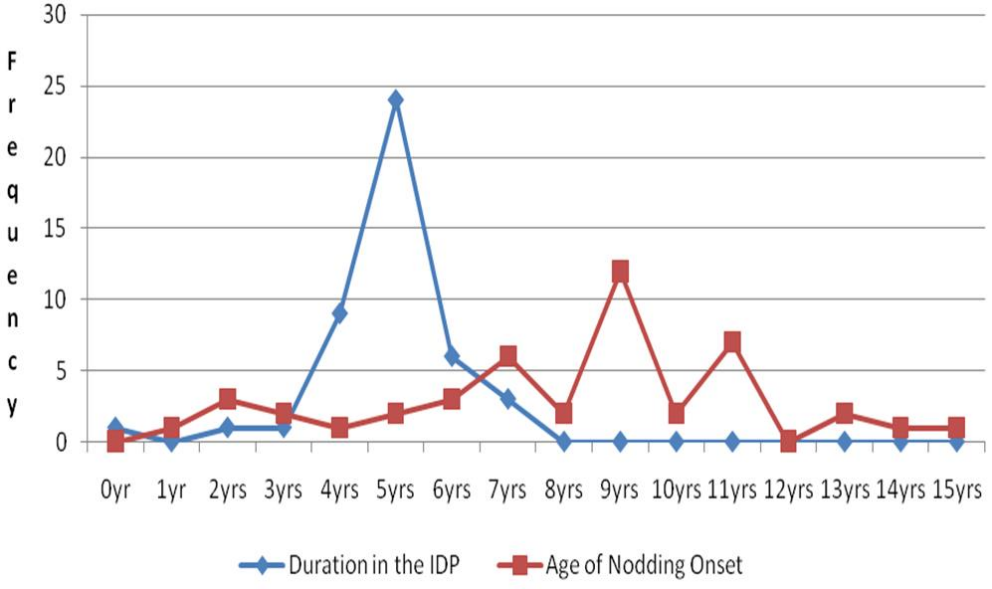
Duration in IDP in relation to age of NS onset (Kitara et al, 2017)

**Figure 3:**
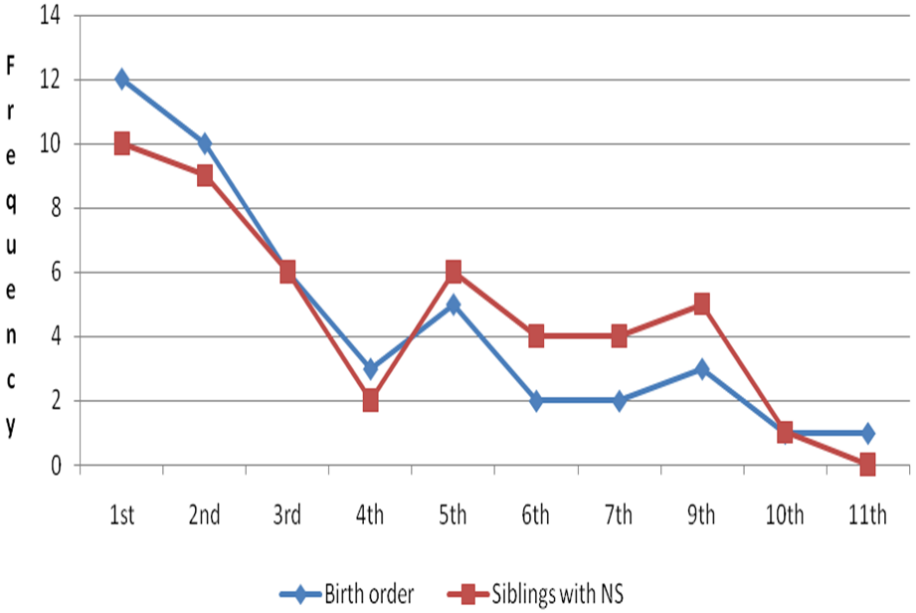
Relationship between NS and birth orders of their NS siblings (Kitara et al, 2017)

The mean age was 14.1 SD ± 2.8years with a minimum of 6 and maximum 19 years (Figure 1). The male to female ratio was 1.5:1 and there was no significant difference (p > 0.05). The mean Body Mass Index (BMI) was 16.9 SD ± 2.7 with a minimum of 11.4 and maximum of 23.2; meanwhile the mean Mid-Upper-Arm-Circumference (MUAC) was 19.9 SD ± 2.8cm. In addition, all NS children were in IDPs (Figure 2) and the majority (77%) had dropped-out of school. The head of households were exclusively peasant farmers and the majority of NS children were in 1^st^, 2^nd^ and 3^rd^ birth order (Figure 3). The number of NS siblings were notably higher in families where the NS child who was 1^st^ (10/45), 2^nd^ (9/45) and 3^rd^ (6/45) born respectively in descending birth order (Figure 3). Urine organic acid analysis: The urate concentration was generally normal (83%); the urate/creatinine ratio was generally low (66%) and the other organic acids were high.

## Discussion

**Figure 4:**
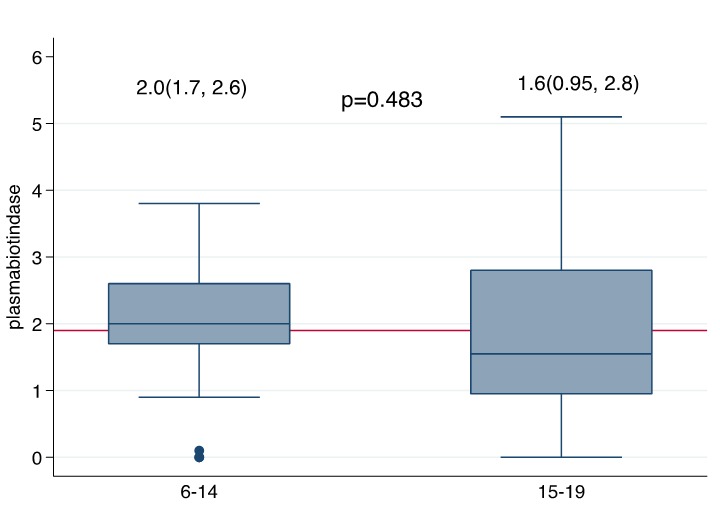
Median plasma biotinidase level by age of NS children

**Figure 5:**
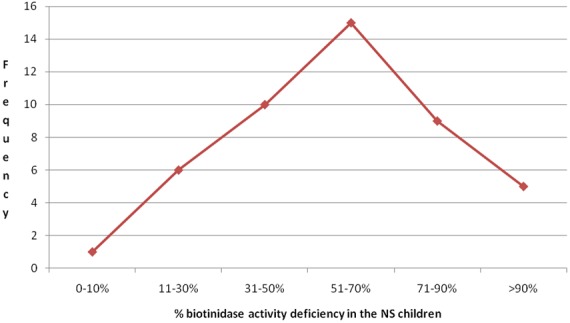
The percentage biotinidase deficiency in NS children

**Figure 6:**
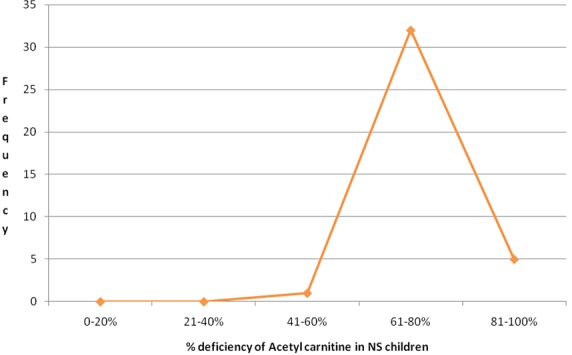
The percentage acetyl carnitine deficiency in NS children

Epidemiological findings: The peak incidences of NS onset were in IDPs (Figure 1). The reported month of peak incidences of NS onset were in April and October which corresponded with the peak monthly average rainfall received for the 1^st^ and 2^nd^ rainy seasons and seasonal deficiency in the availability of food in the region [12]. All NS children experienced IDP life which peaked at 5 years (Figure 2) and were commonest in the 1^st^, 2^nd^ and 3^rd^ birth orders (Figure 3) [12]. Interestingly, most NS children had siblings with NS which mirrored their birth orders (Figure 3) [12]. NS parents had no reported symptoms or signs of NS and the offsprings of some NS patients that delivered 3 years prior to the study were reportedly normal. This finding perhaps suggests an acquired disease which was not transmissible to their offspring. Secondly, this condition could be arising from a family stressor e.g malnutrition and/or infections which were experienced during IDP [12,16]. Perhaps the perfect examples could be seen in deficiencies of metabolites (Figure 4, Figure 5, Figure 6) in acquired diseases which become overtly expressed during stress as we suggest could have occurred in NS [17]. The communities of NS sufferers were of Acholi and Lango ethnicity and in general, the information from parents of NS children show that they were all reportedly born normal and that their developmental milestones were normal until nodding began [9,11,12]. Upon being recruited to the HfH rehabilitation centre and feeding on locally prepared food supplement (MAMA food supplement Ltd), plus anticonvulsants and multivitamins, their health conditions greatly improved, seizure frequency reduced, mental health status and cognitive impairment improved, they gained weight and height and by 2014 when the authors reassessed them, most were categorized as MAM and healthy, nutritionally [10,12]. However, much as they had improved and some had returned to school, none could be declared cured by Ugandan MOH or WHO because they still experienced sporadic episodes of nodding, emotional, perceptual disturbances and cognitive impairments (Table 1, Table 2) [10,11,12]. Interestingly, since 2012 when the IDPs were disbanded and communities returned to their villages and feed on locally grown foods, no new cases of NS had been reported by Ugandan MOH or WHO. Therefore, a disease which is self limiting and occurred only in children that experienced IDPs could perhaps best be associated with the IDPs, diet and environmental factors.

**Table 1:** The bivariate analysis of factors associated with nodding syndrome

Variables	(χ^2^)	p-value	Fisher's test
Sex of NS child (Male)	1.134	0.287	0.245
Age at NS onset	10.218	0.511	0.477
NS child was in IDPs	**22.15**	**0.005**	**0.004**
NS child had other siblings with NS	**9.86**	**0.004**	**0.045**
Length of IDP stay	7.500	0.277	0.277
Birth order of NS child	9.680	0.377	0.270
School Attendance	0.761	0.683	1.000
Caretaker is a mother	**6.392**	**0.041**	**0.140**
>50 nodding episodes since NS onset	**22.146**	**0.005**	**0.296**
Epileptic fits experienced by NS child	**4.635**	**0.099**	**0.180**
Disorientation	1.907	0.385	0.327
Loss of consciousness	**5.756**	**0.056**	**0.155**
Localized Tonic clonic seizures	0.598	0.742	1.000
Generalized Tonic-clonic convulsions	4.186	0.123	0.151
Urinary incontinence	3.139	0.208	0.367
Sleeping after nodding episodes	3.220	0.200	0.252
Confusion after fits/Nodding	4.430	0.107	0.327
Rhythmic jerking during nodding episodes	2.616	0.270	0.236
Good sleep pattern	1.529	0.675	1.000
Aggressive behavior after fits/nodding	2.188	0.139	0.233
Foaming in the mouth	**3.447**	**0.063**	**0.137**
Perceptual disturbances before/after nodding	1.155	0.283	0.410
Presence of visual hallucinations	3.447	0.486	0.384
History of mental illness in the family	**3.205**	**0.073**	**0.212**
Low serum Biotinidase levels	**11.756**	**0.000**	**0.002**
Low serum Acetyl Carnitine levels	**13.346**	**0.000**	**0.004**
Good family social support to NS child	**10.586**	**0.005**	**0.088**

**Table 2:** Multivariable logistic regression analysis of the associated factors of NS

Variables	Mean	(95% CI)	p-value
Low BMI	16.9	16.10,17.75	0.42
Low MUAC	19.9	19.02,20.76	0.38
Duration in IDPs (yrs)	4.8	4.48,5.21	0.92
Low serum biotinidase	**1.98**	**1.61,2.34**	**<0.001**
Low Acetyl Carnitine	**4.68**	**4.02,5.34**	**<0.001**
Age at NS onset (yrs)	8.02	7.03,9.01	0.64
Current age(yrs)	14.08	13.24,14.92	0.77
Normal Urate/Creatinine ratios	0.25	0.20,0.30	0.08
Normal Urate level	0.23	0.20,0.25	0.45

The normal ranges for serum biotinidase is [2.5-7.5IU/L; serum acetyl carnitine [25-54μmol/L in male Children≤17 years and 19-51μmol/L in female children≤17 years; Urate [0.11-0.3mmol/L]; Urate/creatinine ratio [0.3-0.8mmol/L]

**Figure 7:**
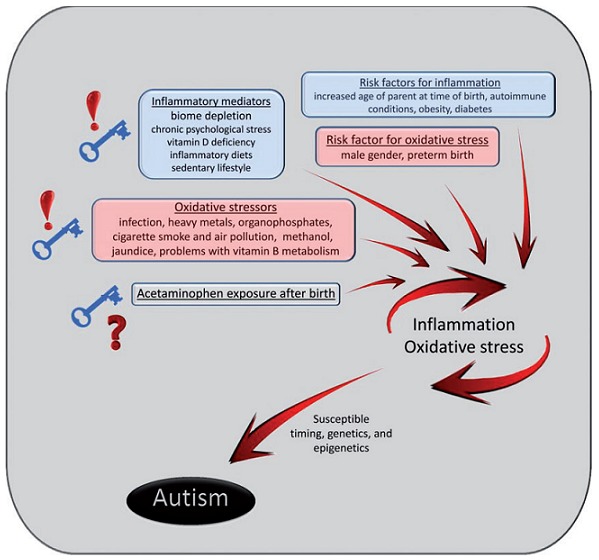
The oxidative stress and possible exposures after birth in the induction of autism

Nodding syndrome, biotinidase and acetyl carnitine deficiency: Most NS children studied had deficiency of biotinidase ranging from 0.0% to 100.0% (Figure 5) (Table 2). The mean % deficiency was 78% (78 SD+13.362). The ranges of deficiency may perhaps be an indication that this was a spectrum which varied considerably from one NS child to another. The clinical features of biotinidase deficiency varies considerably depending on the percentage deficiency [17,18-20]. Biotinidase deficiency has commonly been classified as partial or profound deficiency whereby the clinical presentations depended on the degree of deficiency and presence of stressors [17-20]. The stressors could have perhaps been the IDPs, where there was inadequate food with a resultant malnutrition [11,12,16] or OV infection which afflicted nearly 80% of them [9-13]. Other sources of stress could have been infectious illnesses that were common in IDPs and affected a large number of IDP residents [11,12,21] (Figure 7). If levels of serum biotinidase are low, then biotin cannot be broken down and released from proteins into the diet [17-20]. In addition, biotin serves as a coenzyme for four carboxylases: propionyl-CoA carboylases & β-methyl crotonyl-CoA carboxylases which are important in protein catabolism; pyruvate carboxylases are essential in gluconeogenesis and acetyl CoA carboxylases are involved in the first step in fatty acid synthesis [19,20]. Similarly, most NS children had acetyl carnitine deficiency (Figure 6), a metabolite responsible for the transfer of short chain fatty acids into the mitochondrium for metabolism (Table 3). This perhaps shows that at the time of stress, NS children were unable to utilize short chain fatty acids in mitochondrial metabolism. In addition, a previous study had noted a near significant association with pyridoxine deficiency (Bunga's study (p = 0.06)) [22].This finding was important since seizures are associated with abnormal pyridoxine metabolism [22]. Additionally, it had been observed that NS was associated with vitamin D deficiency [23]. Interestingly, findings in other studies indicate that the levels of organic acid in urine were high and consistent with high anion gap metabolic acidosis observed in a case-control study [9]; case series [23] and clinical studies [12,23]. Therefore, NS in Northern Uganda may perhaps represent an emerging neurological disorder where investigations searching for potential environmental toxins have been extensively conducted but with no uniform identifiable link [10,12]. In addition, NS in south Sudan and Northern Uganda is suspected to be caused by a chemical neurotoxin from war munitions used during the civil war [9,11,12]. However, there are no studies investigating quantifiable war munitions or chemicals as possible causes, although several case control studies have demonstrated associations with exposure to war munitions and gun raids [22]. A recent case series in Northern Uganda found that NS children had been exposed to both severe war-related psychological and physical trauma and that those interviewed laid blame on war munitions/chemicals [24]. These findings suggest that environmental exposures of the affected communities were reported although not proven but could still form a basis for the hypothesis that it could be a factor that could not be ignored in the epidemiology of NS.

**Table 3:** Metabolic disorders associated with epilepsy and autism spectrum disorder

Disorder	Clinical features	Diagnostic testing
*Disorders of energy metabolism*		
Mitochondrial disease	Developmental regression, gross motor delay, fatigability, ataxia and gastrointestinal abnormalities	Fasting serum lactate, pyruvate, acylcarnitine, amino acids and urine organic acids
Creatine metabolism disorder	Developmental regression, mental retardation, dyskinesia, and family history of x-linked mental retardation	Magnetic resonance spectroscopy, Urine and serum creatine and guanidionacetic acid
*Disorders of cholesterol metabolism* Smith-Lemli-Opitz syndrome	Low birth weight, failure to thrive, poor feeding, eczema, and congenital structural abnormalities of the heart, gastrointestinal tract, genitalia, Kidney, limbs, face and brain	Blood 7-dehydrocholesterol and cholesterol, DHCR7 sequencing
*Disorders of cofactor (vitamin) metabolism,* Cerebral folate deficiency	Ataxia, pyramidal signs, acquired microcephaly, dyskinesias, and visual and hearing loss	Folate receptor alpha autoantibody, Cerebrospinal fluid 5-methyltetrahydrofolate
Pyridoxine-dependent and pyridoxine-responsive seizures	Mental retardation, breath-holding, aerophagia, and self injurious behaviour	Pyridoxine trial, plasm and CSF fluid pipecolic acid, urine @aminoadipic semi aldehyde, ALDH7A 1 sequencing
Biotinidase deficiency	Developmental delays, seborrheic dermatitis, alopecia, feeding difficulties, vomiting, diarrhoea, brain atrophy and ataxia	Biotinidase activity, BTD gene sequencing
Carnitine biosynthesis deficiency	Nondysmorphic male–male siblings with autism spectrum disorder	Plasma and/or urine 6-N-trimethyllysine, 3-hydroxy-6-N-trimethyllysine, and gamma butyrobetaine
*Disorders of γ-aminobutyric acid metabolism Succinic Semialdehyde dehydrogenase deficiency*	Global developmental delay, myoclonus, hallucinations, ataxia, choreoathosis and dystonia	Urine gamma-hydroxybutyric acid
*Disorders of pyrimidine and purine metabolism, Adenylosuccinate lyase deficiency*	Global developmental delay, microcephaly, distinct facies, growth retardation, mental retardation, cerebral vermis hypoplasia, brain atrophy, excessive laughter and extreme happiness	Urine and /or cerebrospinal fluid succinyladenosine
Nucleotidase-associated PDD	Hyperactivity, compulsiveness, speech abnormalities, ataxia, abnormal gait, and frequent infections	Urine uridine
Hyperuricosuric autism	Altered sensory awareness, ataxia, and fine motor deficits	24-hour urine urate
Phosphoribosylpyrophosphate synthetase deficiency	Developmental delay and ataxia	Urine uric and orotic acids; Complete blood count
*Disorders of amino acid metabolism,* Phenylketonuria	Global developmental delay, mental retardation, microcephaly, spasticity, ataxia, poor growth, poor skin pigmentation and aggressive behaviour	Serum phenylalanine
Branched-chain ketoacid dehydrogenase, Kinase deficiency	Intellectual disability and consanguinity	Plasma and cerebrospinal fluid branched-chain amino acids
Altered tryptophan metabolism	No specific features besides autism spectrum disorder	Reduced cellular generation of nicotinamide adenine dinucleotide
*Urea cycle disorders*	Protein intolerance, temperature instability, ataxia, episodic somnolence and lethargy, cyclic vomiting and psychosis	Plasma ammonia and amino acids, Urinary orotic acid
*Urea cycle disorder*		

**Nodding syndrome and autism spectrum disorder (ASD)**: Studies on autism spectrum disorder (ASD) show that it is an emerging and dynamic system of metabolic and immune anomalies involving many organ systems, including the brain and environmental exposures [25,26]. To date, it is not yet clear how gastrointestinal (GI) factors are related to ASD [25,26] however, many patients with ASD have a history of previous antibiotic exposure or hospitalization, gastrointestinal (GI) symptoms, abnormal food cravings and unique intestinal bacterial populations, which have been suggested to relate to variable symptom severity [25,27]. ASDs have neuro-chemical changes, neuroinflammation, increased oxidative stress, mitochondrial dysfunction, glutathione depletion and altered phospholipid/ acyl carnitine profiles [25,27] (Table 3). In addition, an author suggests that traditional scientific experimentation is required to verify the hypothesis that enteric short-chain fatty acids may be a potential environmental trigger in some forms of ASD [25-27]. This collaborative developments in systems biology particularly examining the role of microbiome and its effects on host metabolism, immunity, mitochondrial function and gene expression, is reported to hold a great promise in ASD study [25,27]. It is further suggested that the GI microbiome produces an array of bioactive metabolic products capable of entering systemic circulation [25-27]. One author suggested that enteric micro-biome and its metabolic products were dynamic and could be altered throughout an individual's life cycle, particularly during the first 18 months of life [28]. Interestingly, it was reported that the metabolic products from the GI tract microbiome could have profound and dynamic effects on host metabolism, immune function and gene expression which happens in many organ systems including the CNS [29]. Another author recommended that it was important to consider the effects of infant formula versus breastfeeding, a high-calorie Western diet, exposure to antibiotics and disinfectants in humans, animals and plants on the alteration of the human microbiome and its metabolites [19,20,30]. These should be considered a possible source of environmental triggers of many diseases of increasing incidence including ASD [19]. This was particularly evident from human populations who migrated to Western societies, such as the Somalis in the diaspora, who appeared to have a much higher incidence of ASD than it existed in their country of origin [25,31]. Furthermore, there are examples of these experiences in biology to show that it may be possible that a GI biome could alter the behavior of animals [25,32-34]. Examples; Rabies and *Bornavirus* infect the CNS in animals and induce aggression that spreads the virus in the saliva from one animal to another through biting behaviours [25]; Cordyceps (*Ophiocordyceps unilateralis*); a fungal infection that affects the behavior of ants, causing them to climb to the top of plants before they die [25]. The resulting fruiting bodies of the fungus then sprouts out of the dead insect to spread spores [25]; *Toxoplasmosis* causes rodents to act without an appropriate fear response, leading to transmission of the infectious agent through cats via predation and ultimately on to humans [25]; The Mundane acts such as sneezing with common cold or increased gastric motility leading to nausea and vomiting in viral gastroenteritis are suggested to be in the best interest of spreading the infectious agent [25]. The researcher then ponders whether similar things that happen such as carbohydrate craving, diarrhea and fecal smearing in ASD helps to feed and spread bacteria [25]. It was noted that families of ASD children just like NS children often become more alienated when they are told about their children's regressive condition and that there was little that could be done and they are often encouraged to use medications to partially reduce aggressive behavior and to wait for their turn for behavioral intervention programs that take years to begin and to complete [25,26]. This has been observed in parents of NS children who have in their helplessness resorted to using herbal medicines including and not limited to crashed roots, traditional medicines, witchcrafts, prayers, visits to shrines and animal sacrifices as remedies for the treatment of this illness [11,12]. In addition, there are new interesting issues to learn about some observations such as bizarre food cravings, GI symptoms, epilepsy, infectious processes and metabolic disturbances in children affected with ASD [25,35-37] just like NS children. However, there are reports that some ASD children appeared to improve, either spontaneously, after certain broad spectrum antibiotics or possibly by altering their diet [25]. Interestingly, this particular scenario has been observed in NS children at the HfH rehabilitation centre in which NS children whose feeding pattern was changed (using a locally prepared MAMA food supplements) and multidisciplinary treatment have improved physically [10,12]. This researcher suggests that there might be a common digestive system link to these findings even if current understanding in conventional western medicine could do little for ASD and NS children. The mitochondrial disorders observed in ASD-studied at Rossignol Medical Center, California, and Richard Frye, University of Arkansas, appeared to occur largely through environmental and not inherited means [25,38,39].

It is reported that these disorders observed might be caused by or at least worsened by enteric short-chain fatty acids including propionic acid from GI tract bacteria [25,27,38,39]. This is similarly a suggestion being advanced on NS children seen in Northern Uganda and South Sudan because first, they were made to feed on food provided by the relief agencies which were not their usual diet during IDP camps (Plumppy nuts, powdered milk, soya beans, red sorghum, rice and yellow posho and cooking oil). Secondly, there have been consistent observation in case control studies, case series, case reports that NS children have high anion gap metabolic acidosis with depleted bicarbonate levels and one author suggested that the cause of this syndrome may perhaps be due to mitochondrial disorders (Table 3), a factor which may be common between ASD and NS [9,11-13]. Furthermore, the work of Dr. Frye, who reviewed his ASD patient population and found a large subset with the lipid (acyl carnitine) and biochemical (citric acid, glutathione) deficiency (Table 3) are findings predicted by the propionic rodent model was yet another breakthrough in the advancement of science on ASD [25,38-41]. His finding in June 2012 that there was absence of genetic abnormalities to explain these changes suggested that the biochemical findings in ASD stemmed from environmental factors and were not inherited [25,40,41]. These findings were similarly observed in NS children in Northern Uganda where there have been observed Acetyl carnitine and biotinidase deficiency in a pilot study (Table 2, Table 3). In addition, a recently work at New York Medical College, found that short chain fatty acids including propionic acid were histone deacetylase inhibitors and thus was switchers for genes particularly those involved in the metabolism of catecholamines and was important in anxiety, arousal, movement disorder, aggression and craving [25]. Additionally, some researchers now argue that these GI bacteria through natural selection, may be controlling or modulating our behavior and may serve the host well until environmental factors such as Western diet or overuse of antibiotics reset the microbiome to produce alterations of this behavior; the obsessions, perseverations, food fixations and tics but also at times enhanced memory associated with ASD [25,42-44]. It is further reported that propionic and related short-chain fatty acids could elicit behaviors that are anxiety-like, perseverative, repetitive, ritualistic and antisocial behaviour [45-47]. These behaviors were reported to be common to many other neuropsychiatric conditions (obsessive compulsive, mood, anxiety, attention deficit/hyperactive and eating disorders, irritable bowel syndrome, and schizophrenia) where infectious agents have been suggested [25,46]. Another researcher argued that there was a growing incidence of ASD and ASD-related conditions, coupled with the observed alterations in the human microbiome secondary to dietary, medical and agricultural factors and their potential effects on human and animal behavior should be further examined [25,29,46,48,]. Additionally, Professor Jared Diamond contended in his book *Guns, Germs and Steel* that the impact of human migration and urbanization, domestication of plants and animals and resultant human diseases shaping cultures was not trivial [49]. He stated, “It was not so far-fetched to say that Western society has altered human microbial populations, which in turn may be altering human behavior and culture” [49]. The similarities in the clinical presentations and the biochemical findings in children with NS and ASD (Table 3, Figure 7) draws the attention of these researchers to the understanding that NS may perhaps be a condition akin to ASD; a disease spectrum that is not well understood but continues to ravage the lives of many young people and families in developing and developed world. NS were seen only in children who were born normal, lived in IDPs, were from poor families, suffered ranges of infectious diseases, commoner in males, all fed on food ration foreign to their GIT and that all the children who developed NS were IDP residents at some stage in their early lives (Figure 5). The relief agencies distributed various forms of cereals/grains and cooking oil which were perhaps foreign to the GI microbiome of the affected communities and the communities ate them [12]. These factors point to the changes in the diet of NS children and adults in these communities where it occurs at epidemic proportions during and after the IDPs. These factors may have perhaps been partly/or wholly responsible for the syndrome that we have been investigating for many years without finding the cause [10,12]. Important to note was that the Ugandan MOH and WHO have since 2012 reported no new cases of NS when the IDP camps were disbanded and communities returned to their homeland and feed on their locally grown foods. Therefore ASD and NS may be conditions that share many things in common and this may be the right moment to consider them as similar or common entities (Table 3 and Figure 7).

## Conclusion

Nodding Syndrome is a childhood neurological disorder in East Africa and the cause is not known. However, this observational study has demonstrated biotinidase and acetyl carnitine deficiency, which could perhaps lower seizure threshold. Similarly, other studies have demonstrated deficiency in Vitamin B6 and D, high anion Gap metabolic acidosis. In addition, NS children were in IDPs, fed on IDP diets which were mainly foreign to their GI microbiome and other environmental exposures. When the NS children were rehabilitated using home grown food supplement (MAMA supplement plus other symptomatic remedies), their conditions improved and some have returned to school although there are no clear evidence that they have been cured. Interestingly, there are no new cases of NS as reported by Ugandan MOH and WHO since 2012 when the IDP camps were closed and communities resettled in their own communities and feed on their own home grown foods. Although these findings are inconclusive at this stage, NS may be akin to Autism Spectrum Disorder. We recommend a case control study with large sample size to determine the metabolic deficiencies.

**Limitations of this study**: This study was an observational study which was conducted on a limited number of NS patients (47) and some of the information was derived from literature review. In addition, we collected serum and hair samples for further analysis in the biochemical laboratory however, we were unable to complete all amino acid and metal analyses due to resource constraints. Recall bias. The study depended heavily on the accurate information recall from caretakers. All caretakers were living with NS children at the time of nodding onset however, we crossed checked the records that were given by these caregivers at HfH centre and compared with those given in the Government health centres and we found that they were consistently the same.

**Strengths of the study**: This is one of the few observational studies to evaluate the aetiology of this neglected neurological disorder which places the lives of thousands of individuals in East Africa at great risk for life and future. This study was conducted in a well organized rehabilitation centre (HfH) which has been operational since 2012 and most NS children have improved and discharged from the centre although still confronted with emotional, cognitive and perceptual disturbances. The study was conducted in a community in Northern Uganda with a very high burden of NS. Differential participation of individuals with increased disabilities due to prolonged and devastating effects of NS was reduced by reaching out to NS children in the outreaches by travelling to their homes.

### What is known about this topic

- Nodding syndrome is a childhood neurological disorder in East Africa and found in endemic OV areas but clustered in time, space and person;
- Nodding syndrome is associated with cognitive decline, internal displacement and school dropout;
- Nodding syndrome children is associated with metabolic and autoimmune disorders.

### What this study adds

- Nodding syndrome is associated with biotinidase, acetyl carnitine deficiencies and high anion gap metabolic acidosis;
- Some clinical presentations are similar to those of autism spectrum disorder;
- There are potential indications that the NS children experienced oxidative stress during their childhood before onset of nodding.

## Competing interests

The authors declare no competing interests.
